# Responding to the Call: Building a Training Program to Diversify the Academy in Alzheimer's Disease Research

**DOI:** 10.3389/fpubh.2021.671956

**Published:** 2021-06-29

**Authors:** Lucy Annang Ingram, Marvella E. Ford, Christiana L. Johnson, Brianna Ashford-Carroll, Quentin McCollum, Daniela B. Friedman, Sue Ellen Levkoff

**Affiliations:** ^1^Department of Health Promotion, Education, and Behavior, Arnold School of Public Health, University of South Carolina, Columbia, SC, United States; ^2^Department of Public Health Sciences, Medical University of South Carolina, Charleston, SC, United States; ^3^College of Social Work, University of South Carolina, Columbia, SC, United States

**Keywords:** Alzheimer's disease, diversity, mentorship, health disparities, minoritized

## Abstract

Alzheimer's disease and related dementias (ADRD) are at the forefront of the United States (US) public health agenda due to their tremendous human and financial burden. Further, disproportionately high ADRD rates among racial/ethnic minorities require incorporating the unique perspectives of racially and ethnically diverse scientists, which will necessitate diversifying the scientific workforce that investigates disparities in aging. The purpose of this paper is to describe the training and mentorship initiatives of the National Institute on Aging (NIA)-funded Carolina Center on Alzheimer's Disease and Minority Research, emphasizing lessons learned from our engagement with underrepresented minority and minoritized (URM) Scientists. We highlight three aims of the Center's training and mentorship component: (1) Fund pilot projects for URM Scientists conducting research on sociocultural, behavioral, and environmental factors that influence ADRD-related health disparities; (2) Provide mentorship to build the research capacity of Center Scientists; and (3) Offer research education in Health Disparities and Minority Aging Research to Center Scientists and interested researchers at all partner institutions. Our experience may be a practical resource for others developing interdisciplinary training programs to increase the pipeline of URM Scientists conducting ADRD research.

## Introduction

Alzheimer's disease and related dementias (ADRD) have risen to the forefront of the United States (US) public health agenda due to their tremendous human and financial burden (1–3). The overall number of persons with ADRD is increasing due to population aging, with projections of ADRD burden increasing in the US from 5 million adults aged 65 and over in 2014, to about 14 million adults by midcentury (4, 5). Over the same time period, ethnic minorities will become a higher proportion of the aging population. In 2014, 22% of the non-White US population was aged 65 and over, with projections of 45% age 65 and older by 2060 (4).

There is a disproportionate burden of ADRD among racially minoritized communities. For example, the prevalence of ADRD among older Blacks in the US is about 1.5 times higher than older Whites (6), and the number of ADRD deaths among Blacks increased by 99.4% from 1999 to 2004, compared to the 52.6% increase among Whites over the same time period (7). Finding solutions to reducing health disparities requires a racially and culturally diverse group of researchers who can conceptualize what the true problems are from the perspective of diverse populations (8–13). The diversification of ADRD researchers can also lead to an increase in the participation of racially and ethnically diverse populations in ADRD research, as research has found that cultural congruence between the researcher and participant is a facilitator for participation of racially minoritized groups in biomedical research studies (14, 15).

Training programs that increase racially minoritized scholars' aging and ADRD research opportunities are critically important. However, a number of barriers have been noted that have stalled these opportunities and include inequities in training, disparities in levels of research grant support, inadequate program support, limited integration into scientific communities, negative stereotypes about minoritized groups, and implicit bias (16, 17). In fact, a systematic review of mentoring programs for minoritized faculty in academic medical centers concluded that a lack of mentoring might be an important factor in explaining lower rates of success with R01 funding and promotion among minoritized faculty (9).

In 2019, Brewster et al. published a report describing the research priorities that emerged from the National Institute on Aging (NIA)-funded UC Davis Aging and Diversity Conference (10). The experts convened at this conference determined that prioritizing the study of racial/ethnic disparities is essential for achieving equity in healthy aging and dementia care. Additionally, they emphasized that addressing the systems and infrastructure that foster research in aging disparities are important steps toward achieving this equity.

The NIA's commitment to diversifying the research workforce in ADRD is evident in its Health Disparities Research Framework and numerous training programs, including the Research Education Components of its long-standing Alzheimer's Disease Research Centers (ADRCs), AD/ADRD T32 institutional training grants, and most recently, an expansion of their Resource Centers for Minority Aging Research (RCMARs) programs, to include an additional set of Centers focusing exclusively on ADRD. The dual purpose of these AD-RCMARs is to both (1) enhance the diversity of the aging research workforce by mentoring promising underrepresented minority and minoritized (URM) scientists for sustained careers in ADRD-relevant research, and (2) develop infrastructure to increase the number of researchers focused on health and well-being of racially minoritized elders. The RCMAR program is funded through the NIH R24 (Resource-Related Research Project) and P30 (Center Core Grants) mechanisms and awarded to qualified institutions as designated by NIH guidelines. Applicants are required to organize their proposal around a series of core activities related to the Center's infrastructure-building and mentorship goals. Three Cores/Components are required and include an Administrative Core, Research Education Component and an additional Core (e.g., Analysis Core, Community Liaison and Recruitment Core, or other Resource Cores). Applications are evaluated for scientific and technical merit on the basis of the proposal's significance, investigative team, innovation, approach, and environment and in accordance with NIH peer review policy and procedures.

The authors of this article are the recipients of an AD-RCMAR funded in 2017. Our Center addresses a critical health issue among Black populations in the Southeast, and especially in SC, where there are extremely high rates of ADRD (1, 18, 19). The focus of the Center is on population health and the role of sociocultural, behavioral, and environmental determinants on ADRD disparities. Additionally, the Center highlights research that illuminates the pathways by which social, psychological, economic, and behavioral factors affect health in middle-aged and older adults. The purpose of this paper is to describe the training and mentorship initiatives of the Center. The infrastructure of the Center may be helpful for other interdisciplinary training programs designed to increase the pipeline of URM Scientists who are conducting ADRD research.

## Focus on Underrepresented Minority and Minoritized Scientists

In the US, URM [defined as Black or African American, Hispanic or Latino, American Indian or Alaska Native, and Native Hawaiian and other Pacific Islander (20)] faculty represented only 24% of all faculty at post-secondary institutions in 2017 (21). Similarly, there is limited diversity among faculty at major research institutions in South Carolina. As of 2019, 78.1% of full-time (tenured, tenure track, and not on tenure track) faculty members at South Carolina public universities were White. Approximately 68.4% of tenure-track faculty identified as White; only 8.6 and 3.5% of tenure-track faculty identified as Black/African-American or Hispanic/Latino, respectively (22). Additionally, researchers from racially/ethnically diverse backgrounds have been less likely to receive NIH R01 funding. For example, during fiscal years 2000 through 2006, among R01 grant applicants who have doctoral degrees and are working at U.S. institutions, Black researchers were 10% less likely to be awarded R01s compared with White counterparts, even after controlling for their country of origin, educational background, training, past research awards, publications, and employer (23). Given these data and the need to develop a pipeline of URM scientists to conduct high-impact ADRD research in our State and region, our mission is to increase the diversity of the research workforce focused on population health and sociocultural, behavioral, and environmental determinants of ADRD disparities. We accomplish this through sustained infrastructure that supports URM postdoctoral fellows and junior faculty members through a comprehensive and interdisciplinary training and mentoring approach.

## Center Infrastructure: The Learning Environment

**Figure 1:**
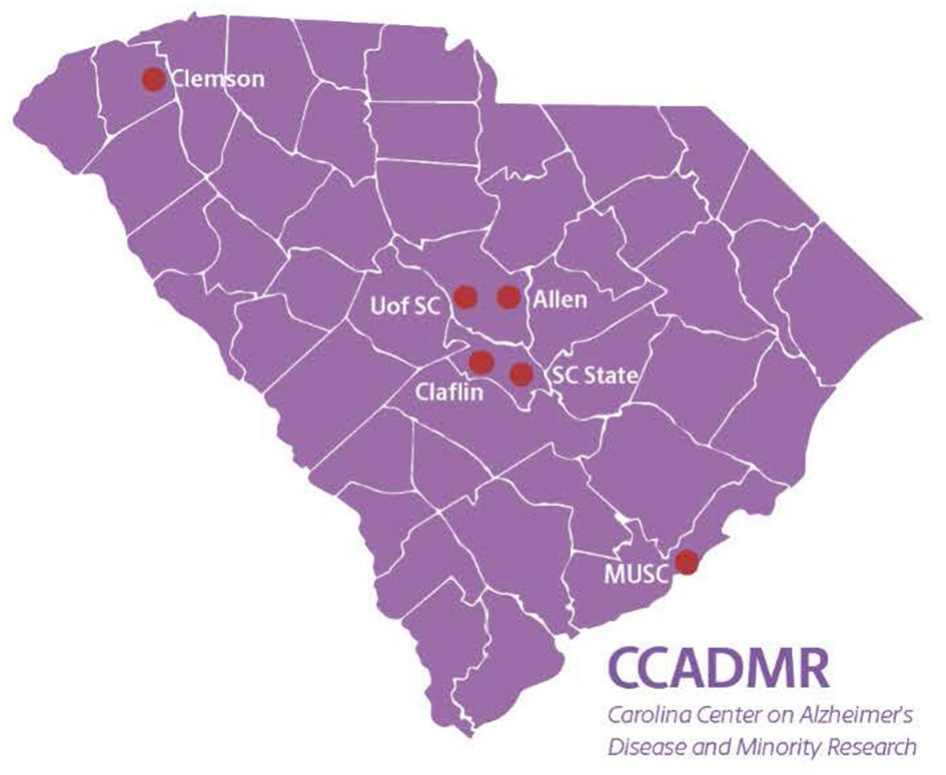
Academic affiliations of the Carolina Center on Alzheimer's Disease and Minority Research.

The Center is part of the national NIA-funded RCMAR network. The network is comprised of 10 traditional Centers, eight Alzheimer's-focused Centers, and a Coordinating Center. Our Center is a statewide collaboration between a large state-funded University, three Historically Black Colleges and Universities (HBCUs) in the State as well as a medical University and a large land-grant University. All of these institutions are in close geographical proximity, with two universities located in Columbia, SC, two universities located in Orangeburg, SC, and one University located in Charleston, SC. All of the universities are within 3 h driving time of each other. See Figure 1 for a map of these academic institutions in South Carolina. All participating institutions have established relationships, some through other funded grants and prior network affiliations. Each partner institution has a site principal investigator (PI) who serves on the Center Executive Committee that oversees the plans of the Center to ensure research education and mentoring activities are of the highest scientific and ethical standards.

The Center consists of an Administrative Core, a Research Education Component (REC), and an Analysis Core. The REC is designed to recruit, train, and establish a mentorship network for a talented cadre of URM faculty at participating institutions who are committed to becoming independent investigators in advancing the science of ADRD through population-based research. Below, we highlight three aims of the REC that include: (1) Funding pilot projects for URM Scientists who will conduct population-based research to advance research on sociocultural, behavioral, and environmental factors that influence ADRD-related health disparities; (2) Providing mentorship to build the research capacity of Center Scientists; and (3) Offering research education in Health Disparities and Minority Aging Research to Center Scientists and interested scholars at all partner institutions.

### Funding Pilot Projects for URM Scientists

The Center REC Team, with the support of the Center Executive Committee, developed a Call for Proposals that is disseminated electronically to the Center network. The Call is typically distributed in the Fall, with an application deadline in early Spring, and a project start date of the following Fall. Our network includes Center Team members (Center MPIs, Core Leads, Partner Institution PIs, and Center Staff) as well as persons who have attended Center events and shared their contact information to be notified about Center events. We have found that a snowball-type effect of using this core network to then distribute the announcement among their professional networks has been most successful in garnering strong, competitive applications for the pilot funding opportunity. Additionally, each partner institution's site PI is charged with personally contacting faculty and/or post-doctoral fellows at their home institutions who might be interested in the funding opportunity. The maximum period of award per pilot project is 12 months for up to $30,000 in total costs.

The application includes a three-page research plan, a letter of support from a faculty colleague from the applicant's home institution who is familiar with the applicant's research, as well as administrative forms, including the NIH face page signed by an authorized institutional official, a description of the project/performance sites/key personnel, detailed budget and justification, biographical sketches of key personnel, and resources page. Applications must focus on investigations using secondary datasets [i.e., the SC Alzheimer's Disease Registry, the Health and Retirement Study (HRS), or the Behavioral Risk Factor and Surveillance System (BRFSS)] for research on the sociocultural, behavioral, and environmental determinants of ADRD health disparities. For this reason, applicants are required to communicate with a member of the Analysis Core prior to submission to ensure feasibility of their proposed investigation.

The application review criteria are based on NIH review criteria (i.e., overall impact, significance, investigators, innovation, approach, environment) as well as the applicant's potential for obtaining future extramural funding, and budget. Each pilot project application is sent to three mid- to senior-level Center faculty members with expertise in the subject matter of the proposal. One of these three reviewers includes a member of the Analysis Core. While there is a primary reviewer designated for each proposal, each reviewer scores the assigned proposal based on review criteria using an NIH format and scoring criteria. Then, all reviewers meet in an NIH-style study section virtual meeting to discuss their critiques of each proposal. Discussions for each proposal are led by the designated primary reviewer who provides initial critiques, followed by the other assigned reviewers and review panel. After the discussion, final scores are taken. The top-ranked proposals are recommended for funding and submitted to NIA for their final review and approval. In some cases, proposed investigators are asked to make minor revisions to their proposals before they are submitted to NIA for approval. All applications are reviewed, with critiques sent to both funded and unfunded applicants. Persons who are not recommended for funding are encouraged to re-apply for future application cycles.

To date, nine pilot project proposals (three per grant year) have been funded, as was the goal of the Center. Ninety percent of the Scientists are female, 56% are Black, 11% are Asian, 11% are Latinx, and all have terminal degrees (90% of which are PhD) with other credentials and certifications including APRN, AGPCNP-BC, and MPH. Brief written progress reports from the pilot project principal investigators are required quarterly. Presentations of the work completed to date are required at the end of the project year. The proposal acceptance rate is 43% for each year that we have had an open call for proposals.

### Providing Mentorship Opportunities

The evidence is clear about the extreme value that mentorship can have on increasing faculty diversity in the sciences, promoting retention of minoritized faculty in academic settings, and enhancing productivity in conducting research (9, 11, 24–27). Particularly in cases where URM faculty may work at institutions where the research infrastructure is limited, the climate is stifled, or the environment is not conducive to supporting their research scholarship, promulgating a supportive environment through dedicated, effective mentorship can be incredibly beneficial to the success of minoritized faculty in academia and help to propel their professional trajectories. In fact, research has shown that, to mentor effectively requires constructing environments that promote self-efficacy and affirm individual identities, particularly for diverse, URM scholars (28). Our mentorship approach is guided by this philosophy and is supported by two mentoring frameworks: the mosaic model and the multiple mentor model.

The mosaic model is a culturally responsive mentoring program that was designed for faculty and staff of color to foster support and interdependence (29). While focused on assisting University faculty and staff to navigate University infrastructure, we find that its tenets of multidimensional guidance and potentially long-term career mentoring are applicable to our approach to providing a support network for our Scientists. Further, the multiple mentor model is one in which multiple mentors work with a single mentee, therefore enhancing the opportunity for mentorship to be adapted to the needs of the mentee and further extending their potential for career success (28).

Applicants who are selected to receive the Center award for pilot research are accepted into the program and referred to as Center Scientists, a term agreed upon by the NIA Coordinating Center. Upon selection and invitation to be part of the Center, Scientists are asked to complete a Scientist Agreement form that outlines information about milestones, requirements, timelines, and expectations. Items include developing a career development plan, submitting quarterly progress reports, attending monthly Center seminars/meetings, developing a manuscript based on pilot project findings, and tracking their progress and productivity for up to 5 years for evaluation purposes. It is expected by Center leadership that each funded Scientist produces a publishable manuscript and both attends and presents their progress at Center meetings.

Each Scientist is assigned three Mentors. Potential Mentors are discussed during the application review process and are selected based on the proposed work of the Scientist to enhance their aging, ADRD, methodological, and health disparities knowledge base. Mentoring teams are interdisciplinary, typically with one Mentor having research experience in the social and behavioral aspects of ADRD and the others with expertise in methods/analysis or another subject area relevant to the Scientists' proposed work.

Potential Mentors are mid-career/senior faculty from the partner institutions who respond affirmatively when approached by Center leadership about becoming a research Mentor for the specific Scientist. The leadership then provides the prospective Mentors with information describing mentorship responsibilities and expectations. These include participating in regular meetings with Scientists to help inform their pilot projects, guiding Scientists on the development and implementation of their career development plans, and participating in quarterly meetings with Center leadership to discuss Scientist progress. Similar to the Scientists, Mentors are also asked to complete a Mentorship Agreement outlining their role and responsibilities as part of the mentorship team.

Once prospective Mentors have been identified and confirmed, Scientists and Mentors are introduced by the REC leadership and they discuss their preferred methods of meeting/communication. Center leadership encourages teams to begin with the Scientists' career development plan to initiate the relationship. It is important to note that while Scientists are assigned initial Mentors upon pilot award funding, opportunities to restructure or add to their mentoring group are encouraged as needed. Every 3 months during the 12-month period of pilot project implementation, Center leadership meets with Scientists and Mentors separately, in order to better assess the Scientist-Mentor relationship, provide suggestions for additional support, and/or determine opportunities to enhance the relationship. An external evaluator also interviews each Scientist and Mentor to learn about their experiences with the goal of improving overall Center and REC processes.

### Offering Research Education in Health Disparities and Minority Aging Research

To implement this aim, we developed the Center seminar series focused on ADRD health disparities, aging among racially minoritized groups, cognitive aging, population-based data, and professional development. After brainstorming potential topics/speakers at monthly steering committee meetings, and taking into consideration feedback from participants from previous seminars, we formulate a slate for the upcoming year. Topics have ranged from a specific ADRD and aging focus such as “Overview of health disparities in aging and ADRD” to a professional development focus such as “My journey to an academic position” to a more methodological and analytic focus such as “Interdisciplinary approaches to geriatric care and the analysis of relevant data.” See Table 1 for a full listing of seminar topics and speakers.

**Table 1:** CCADMR Health Disparities & Minority Aging Research Seminar Series (2019–2020).

**Date**	**Topic**	**Speakers**
February 15, 2019	CCADMR Health Disparities & Minority Aging Research Education Seminar Series Kickoff	CCADMR Leadership
March 8, 2019	Introduction to the South Carolina Alzheimer's Registry and the Health and Retirement Study	Dr. James Hardin Dr. Maggi Miller Dr. Katrina Walsemann
April 19, 2019	Overview of Health Disparities in Aging and ADRD	Dr. Daniela Friedman Dr. Mindi Spencer
May 10, 2019	Social Determinants of Health in Aging Research	Dr. Ye Luo Dr. Maggi Miller Dr. Katrina Walsemann
June 14, 2019	2018-2019 CCADMR Pilot Research Grant Awardees: Project Updates	Dr. Nicole Davis Dr. Miriam Evans Dr. Andrea Henderson-Platt
July 12, 2019	Interdisciplinary Approaches to Geriatric Care and the Analysis of Relevant Data	Dr. James Hardin Dr. Donna Ray
August 30, 2019	Professional Development	Dr. Marvella Ford
September 13, 2019	Aging Policy	Ms. Taylor Wilson
October 18, 2019	CCADMR Information Session	CCADMR Investigators and Scientists
November 8, 2019	Engaged Aging Dementia Care and Research and Community Outreach	Dr. Cheryl Dye Ms. Caitlin Torrence
January 24, 2020	How to Write A Data Analysis Section	Dr. James Hardin
February 14, 2020	Updates from Year 2 Scientists CCADMR Scientists	Mentors
March, April, May 2020	POSTPONED	POSTPONED
June 12, 2020	Research Collaborations: Tips, Tactics and Tales	Dr. Daniela Friedman
July 24, 2020	Updates from Year 2 Scientists CCADMR Scientists	Mentors
September 11, 2020	Understanding Dementia	Ms. Megan Byers
Oct. 9, 2020	CCADMR Fall Social CCADMR Scientists	Mentors
November 13, 2020	Regional Alzheimer's Registries	Dr. Maggi Miller Ms. Rana Bayakly

Seminars were originally offered monthly in both live and virtual formats, due to the geographical spread of our partner institutions. In March 2020, we were able to successfully transition to live streaming only due to the COVID-19 pandemic. When live seminars were held, the majority were hosted by, and located at the University of South Carolina; however, several seminars were hosted at partner institutions to facilitate access for scholars across the State. This allowed for face-to-face networking. All seminars are recorded and archived on the Center website for later viewing (https://sc.edu/study/colleges_schools/socialwork/research/ccadmr/research_education/hdmar/). The video conference platform, Zoom, is used to live stream and record seminars. The use of the Zoom platform to livestream the seminars allows attendance to easily be taken as persons are required to log in, and thus identify themselves, upon entry onto the host site for the seminar. Zoom was also chosen because it is accessible by participants at all of the partnering institutions.

Evaluation of the seminar series allowed participants to provide feedback on a number of measures, ranging from satisfaction with seminar content and speakers, to more cognitive level processes such as organization of thoughts/ideas, increased knowledge and comprehension of the topic of interest, and potential actions that the seminar could spur such as sharing the information learned with one's broader network or following up with the speaker for more information or a potential collaboration. Evaluation questions are primarily closed-ended; however, open-ended questions provide respondents the opportunity to add additional feedback about recommendations for future seminars or other information that they would like to share. The survey was initially developed internally by the Center evaluator as a paper-and-pencil instrument and has evolved to a digital form (hosted by Survey Monkey) that attendees can access via QR code or web address.

Seminar evaluation data, compiled from June 2019-January 2020, has shown that nearly 81% of attendees (who completed the survey) reported that they were very satisfied with their overall experience. This remains true for attendees when asked about each of the seminar speakers (85%), as well as the content presented (84%). The most recent Center seminars that were held virtually (June 2020-November 2020) also revealed similar successes, with about 75% reporting a “very satisfied” rating for the overall seminar experience, and over 90% of attendees being “very satisfied” with the speakers and content.

## Overall Evaluation Plan

The evaluation plan for the aforementioned aims (i.e., funding pilot projects, providing mentorship opportunities, and offering research education in health disparities and minority aging research) is designed to ensure continuous monitoring and assessment of outcomes. For example, we have developed a digital tracking system to assess Scientist productivity based on their quarterly self-report on measures such as conference abstract submissions, manuscript submissions, and progress toward specific aims of their proposed pilot projects. Scientists meet quarterly with REC leadership to report on their pilot project progress as well as on the success of the Scientist-Mentor relationship. These meetings yield a qualitative account of the mentorship relationship and offer opportunities mid-year to make adjustments should changes need to be made.

Additionally, individual qualitative interviews are conducted with all Scientists and Mentors at the end of the pilot project funding year. Interviews are conducted by an external evaluator to discuss Scientist progress toward independent investigator status, and to identify potential challenges and facilitators to overcoming barriers to progress. After their initial pilot project funding period, Scientists are contacted annually to update progress on these measures. Related to the Center Health Disparities and Minority Aging Research seminars, participants are asked to complete an evaluation after each seminar to identify strengths, challenges, and suggestions for improvements.

## Discussion and Lessons Learned

Partner-engaged approaches to planning, implementing, and evaluating REC processes and activities have been critical for the success of the Center and our Scientists. Meeting monthly as a steering committee with all partner institution PIs and Center Core leaders and PIs, many of whom serve as Mentors, and meeting monthly with Scientists and Mentors separately has helped us understand the needs and interests of both Scientists and Mentors. This process has also provided an important opportunity for iterative development of REC initiatives, including seminar topics, wording of the Call for Proposals, and Mentor requirements. For example, through the process of iterative development, we learned that requiring pilot project applicants to communicate with Analysis Core members in advance of submitting an application tremendously strengthened the quality of the applications that were received.

We have also realized the importance of having the Scientists engage and learn from each other, and we plan to increase this opportunity moving forward through formal seminars and more informal gatherings. There is tremendous benefit to the Scientists' growth as AD researchers and colleagues through dialogue about development of research aims, data acquisition and analysis, dissemination, and resource availability, as well as the sharing of professional and career-related questions and issues that arise. This type of learning exchange and peer mentorship is a collaborative process involving a reciprocal relationship that provides opportunities for growth based on common interests (30, 31). As opposed to a more traditional model in which the senior faculty member guides the junior faculty member, this peer or mutual mentorship approach offers bi-directional support for Scientists' professional and personal challenges (32). This learning exchange has shown to be effective especially for identity-concordant relationships and can help improve diversity and inclusion-focused initiatives in academic settings (33).

Also, we learned from partner institution PIs that limiting budget support to graduate students is unintentionally punitive to applicants from some institutions who may not have a significant pool of graduate students matriculating on their campuses. Having partner institutions collaborate on the promotion of seminars and the Call for Proposals has helped increase the number of attendees at seminars and increase the number of proposal submissions from applicants across the Center institutions.

In light of the COVID-19 pandemic, the annual RCMAR in-person meeting was canceled in Spring 2020. While there have been opportunities for Scientists to engage with funders through webinars hosted by the RCMAR Coordinating Center and other funded RCMARs, this virtual time has reminded us of the importance of being creative in how we engage Scientists, Mentors, and Center stakeholders. We are considering additional ways for Scientists to engage with each other and have opportunities for peer mentorship, such as informal virtual discussions and check-ins.

## Conclusion

The research workforce in ADRD research includes insufficient representation of investigators from diverse racial and ethnic backgrounds. This paper presented the key components of a research training program designed to address this problem of underrepresentation. While the evaluation of program outcomes is ongoing, we believe that the operationalization of the key program elements will contribute to long-lasting improvements in the diversity of the aging research workforce. Our experience may be a practical resource for others developing interdisciplinary training programs to increase the pipeline of underrepresented scholars conducting ADRD research.

## Contribution to the Field

The number of persons with Alzheimer's disease and related dementias (ADRD) is growing, with projections of ADRD burden increasing in the US from 5 million adults aged 65 and over in 2014, to 14 million adults by midcentury. ADRD prevalence among Black people in the US is 1.5 times higher than Whites, with Hispanic Americans projected to have the largest increase in ADRD among racial/ethnic groups due to population growth. Given this disproportionate burden of ADRD among minority communities, training programs that increase minority scholars' aging and ADRD research opportunities are critically important. Experts have determined that prioritizing the study of racial/ethnic disparities is essential for achieving equity in healthy aging and dementia care. Furthermore, addressing the systems and infrastructure that foster research in aging disparities are important steps toward achieving this equity. In response, our paper describes the training and mentorship initiatives of the National Institute on Aging (NIA)-funded Carolina Center on Alzheimer's Disease and Minority Research, emphasizing lessons learned from our engagement with underrepresented minority (URM) scientists. Our experience may be a practical resource for others developing interdisciplinary training programs to increase the pipeline of URM Scientists conducting ADRD research, with the ultimate goal of developing interventions to reduce ADRD disparities in the US.

## Data Availability Statement

The original contributions presented in the study are included in the article/supplementary material, further inquiries can be directed to the corresponding author/s.

## Author Contributions

LI, MF, DF, and SL contributed to the conception and design of the study and wrote sections of the manuscript. CJ performed the literature review, wrote sections of the manuscript, organized the manuscript revisions, and technical submission requirements. BA-C and QM gathered data relevant to the study. All authors contributed to the article and approved the submitted version.

## Conflict of Interest

The authors declare that the research was conducted in the absence of any commercial or financial relationships that could be construed as a potential conflict of interest.
